# “Remarkable solutions to impossible problems”: lessons for malaria from the eradication of smallpox

**DOI:** 10.1186/s12936-019-2956-y

**Published:** 2019-09-23

**Authors:** Justin M. Cohen

**Affiliations:** Clinton Health Access Initiative, 383 Dorchester Ave, Suite 400, Boston, MA 02127 USA

**Keywords:** Malaria, Eradication, Elimination, Smallpox, History

## Abstract

**Background:**

Malaria elimination and eventual eradication will require internationally coordinated approaches; sustained engagement from politicians, communities, and funders; efficient organizational structures; innovation and new tools; and well-managed programmes. As governments and the global malaria community seek to achieve these goals, their efforts should be informed by the substantial past experiences of other disease elimination and eradication programmes, including that of the only successful eradication programme of a human pathogen to date: smallpox.

**Methods:**

A review of smallpox literature was conducted to evaluate how the smallpox programme addressed seven challenges that will likely confront malaria eradication efforts, including fostering international support for the eradication undertaking, coordinating programmes and facilitating research across the world’s endemic countries, securing sufficient funding, building domestic support for malaria programmes nationally, ensuring strong community support, identifying the most effective programmatic strategies, and managing national elimination programmes efficiently.

**Results:**

Review of 118 publications describing how smallpox programmes overcame these challenges suggests eradication may succeed as a collection of individual country programmes each deriving local solutions to local problems, yet with an important role for the World Health Organization and other international entities to facilitate and coordinate these efforts and encourage new innovations. Publications describing the smallpox experience suggest the importance of avoiding burdensome bureaucracy while employing flexible, problem-solving staff with both technical and operational backgrounds to overcome numerous unforeseen challenges. Smallpox’s hybrid strategy of leveraging basic health services while maintaining certain separate functions to ensure visibility, clear targets, and strong management, aligns with current malaria approaches. Smallpox eradication succeeded by employing data-driven strategies that targeted resources to the places where they were most needed rather than attempting to achieve mass coverage everywhere, a potentially useful lesson for malaria programmes seeking universal coverage with available tools. Finally, lessons from smallpox programmes suggest strong engagement with the private sector and affected communities can help increase the sustainability and reach of today’s malaria programmes.

**Conclusions:**

It remains unclear whether malaria eradication is feasible, but neither was it clear whether smallpox eradication was feasible until it was achieved. To increase chances of success, malaria programmes should seek to strengthen programme management, measurement, and operations, while building flexible means of sharing experiences, tools, and financing internationally.

## Background

The World Health Organization (WHO) has called for the eradication of malaria since 1955 [1], when the Global Malaria Eradication Programme urged countries to seek to interrupt transmission through an effort involving campaigns of indoor spraying of insecticide on a “total coverage basis” [2]. Although the 1969 World Health Assembly “re-examined” that goal [3], it was never officially abandoned. Today, the WHO’s Global Technical Strategy [4] calls for gradual progress towards eventual global eradication, defined as the “permanent reduction to zero of the worldwide incidence of infection caused by human malaria parasites as a result of deliberate activities” [5]. To reach this goal, the WHO targets having at least ten new countries achieve malaria elimination—defined as the “interruption of local transmission (reduction to zero incidence of indigenous cases) of a specified malaria parasite in a defined geographical area as a result of deliberate activities” [5]—by 2020, ten more by 2025, and another 15 by 2030. Achieving these targets with the imperfect, impermanent tools available to malaria elimination programmes will require internationally coordinated approaches, high degrees of engagement from politicians and communities, efficient organizational structures, and well-managed programmes. As governments and the global malaria community seek to overcome the associated challenges, their efforts should be informed by the substantial past experiences of other disease elimination and eradication programmes.

Smallpox is the only infectious disease of humans to have been eradicated globally. The Intensified Smallpox Eradication Programme of 1966–1977 was a global effort to conduct mass vaccination in combination with surveillance to detect cases and control outbreaks [6]. Dr. Donald A. Henderson, the director of the WHO-led campaign, recounted that smallpox eradication “proved to be infinitely more difficult than I or anyone else had imagined it would be” [7], and believed that the success against smallpox could not be replicated against malaria with the currently available tools [8].

Given that malaria eradication remains the stated global goal, however, it is worth examining the smallpox eradication experience to understand what lessons can be learned about how to approach such an ambitious undertaking. Drawing lessons for malaria from the smallpox programme is inherently challenging due to the different disease dynamics and interventions (i.e., a highly protective, long-lasting vaccine for smallpox *versus* imperfect anti-vector and parasite tools for malaria that must be repeatedly re-distributed). Yet it is plausible to expect there are also political, operational, financial, and administrative commonalities to any global undertaking of this nature. The need to coordinate stakeholders across endemic countries, to finance a long-term enterprise, and to ensure countries act in concert to minimize importation from neighbors means the malaria community will be “forced to navigate complex administrative and societal terrains, where knowledge gleaned from scientific and medical journals can only be partially useful” [9], but where past experiences managing similar programmes may prove valuable.

## Methods

To investigate potential lessons for malaria in the smallpox literature, PubMed was searched on 7 Feb 2017 for “smallpox” and “eradication” in the title or abstract of publications. Seven hundred results were returned. Abstracts were evaluated to assess whether the publication would provide information about how smallpox programmes overcame seven challenges that malaria eradication will likely face, selected in collaboration with members of the WHO’s Strategic Advisory Group on Malaria Eradication. These challenges included the international issues of (1) how support for the undertaking was fostered internationally, (2) how the campaign was internationally coordinated, and (3) how it was financed. At a national level, issues included (4) how support was fostered nationally and (5) at community level, (6) what programmatic strategies were found to be most successful, and (7) how national elimination programmes were most effectively structured and managed. Additional references cited in the PubMed results that seemed relevant, including several books, were also included in review.

Abstracts of all results were examined for relevance to one of these seven areas of interest. The full texts of all relevant publications were read by the author, who noted and collated any information provided on how smallpox programmes addressed these diverse challenges. The potential ramifications or lessons for malaria eradication were evaluated by the author based on his experience providing operational, technical, and financial support to numerous malaria elimination programmes over the past decade in his role as the director of a malaria programme at a non-governmental organization.

## Results

Of 700 results returned by the PubMed search, 118 publications were selected for full-text review. These documents, which ranged in publication year from 1959 to 2015, included contemporary accounts of the campaign from specific countries, assessments of global programme process, and reflections on the eradication accomplishment by its participants in both journal article and book form, along with several reviews of the smallpox experience. A number of themes emerge from the literature across the seven areas of interest (Table 1).

**Table 1:** Summary of challenges faced by the smallpox eradication, factors described in the published literature that enabled programmes to overcome them, and potential lessons for malaria eradication

Theme	Smallpox challenges	Smallpox success factors	Lessons for malaria
International support	Lack of global political endorsement	Backing from major global superpowers Strong, well-connected central leader	Need to maintain public relations and advocacy for campaign
	Limited profile and resources	Engaging national leadership at World Health Assembly Widely released report on progress and challenges	Maintain World Malaria Report as progress tracker and opportunity for visibility Use global forums to hold political leadership accountable
International coordination	Low quality products with stability issues at start of campaign	Clear quality standards and reference labs for quality testing	Maintain global quality assurance structures for malaria products
	Unforeseen challenges necessitated ongoing innovation and research	Decentralized innovation, including development of new tools and testing of new strategies, encouraged by the WHO	Continue investing globally in research and development, while supporting countries to iteratively improve programmes based on quality data and analysis
	Insufficient donor funds to donate vaccine to large-volume countries	Local manufacturing of vaccine in large-volume countries	Consider building local manufacturing capacity for high volume commodities like bed-nets
	Lack of coordination or synchronization between regionally connected countries, and limited impact of international declarations	Small financial incentives to encourage country participation Technical and logistical support staff embedded in country programmes to help build efficient programmes following best practices Embrace independent actions by countries as a way to test many approaches simultaneously across different sociocultural and epidemiological contexts	Use funding to encourage participation Encourage countries to try context-appropriate strategies while encouraging uptake of proven best practices Provide countries with embedded advisors to build both technical and operational/logistical capacity
	WHO’s bureaucracy limited speed and agility of programme, and disparate views on approaches persisted between different levels of the WHO	WHO staff took flexibility into their own hands to quickly respond, circumventing institutional rules and leveraging backchannels wherever possible	Reduce bureaucracy in global and regional coordination mechanisms to ensure flexibility and nimbleness
Financing	Resources were being used inefficiently in country	Transitioning existing domestic resources to more effective management schemes to achieve greater impact without substantial budget increases	Ensure optimal allocation of available funding and strong management and measurement to increase and demonstrate its impact
	Interest in allocating funds to the programme waned with decreasing case counts	Agreement that eliminating in low resource countries would reduce prevention costs in high-resource countries	Continue advocacy efforts, including through business and political champions, to keep malaria a priority even as visibility wanes
	Bureaucratic processes or insufficient funds created bottlenecks in paying staff and transport costs	Flexible funding accounts and reimbursement mechanisms	Increase availability of small but flexible funding that can be used to address bottlenecks across countries as they arise
National support	Competition with other disease priorities limited support, particularly as burden diminishes or when less virulent strains were common Government leadership turnover often led to loss of prior political support	Identification of politically-connected domestic champions Engagement of private sector actors	Build external national support outside government, including identification of malaria champions Create private sector partnerships to maintain elimination enthusiasm and support implementation
Community support	Mistrust of vaccination due to real or perceived adverse events	Engaged or combined mobilization and awareness efforts with other community initiatives (neonatal care, census taking, market days) Gained community acceptance through proactive engagement with community leaders	Conduct community research to understand how to most effectively build support and engagement from affected populations
	Single disease focus may have reduced community participation	Used financial incentives in the endgame to keep up engagement and enthusiasm Created private sector partnerships to extend vaccination and education efforts	Tie malaria elimination efforts to larger health system initiatives (childhood illness, community health, vector control) to increase participation
	Compulsory vaccination approaches elicited negative reactions from community	Discouraged compulsory vaccination	Increase outreach at local level to village leaders to ensure community buy-in and cooperation
Programmatic strategy	Transmission persisted in unvaccinated populations despite high overall vaccination rates	Shift from national mass vaccination to surveillance and focused vaccination in the areas where smallpox was observed Case finding intensified during the period of lowest seasonal incidence, the weakest point of the transmission cycle Global guidance updated and disseminated by WHO as evidence accrued of what worked best	Focus on targeting prevention and treatment to the places where they are most necessary, rather than only evaluating the number of people receiving them Understand malaria seasonality and ensure interventions are intensified at the time of the year when they will be most impactful Continue to update technical and operational guidelines and encourage countries to adopt proven approaches
	Disease reporting depended on independent statistical units and other health system entities not within the control of the smallpox programme	Follow up and routine feedback to all reporting points to ensure good participation Developed a network of agents who conducted active case detection activities Integrated reporting from both health facilities and active surveillance to leverage strengths of both	Provide routine feedback and supervision to all reporting points to ensure high quality data Augment routine reporting from health facilities with active surveillance designed to identify areas of transmission that may otherwise be missed
National programme structure and management	Limitations of existing health system to achieve necessary surveillance and vaccination coverage of at-risk communities, but inefficiency and unsustainability of a fully vertical program	Vertically managed and measured programmes were integrated with basic health systems, allowing smallpox-specific programmes to leverage horizontal systems for surveillance and support	Leverage basic health systems for routine case management and ongoing surveillance Integrate malaria elimination into the health system to improve functionality and cost-effectiveness, while maintaining vertical elements to facilitate fundraising, community mobilization, and political buy-in
	National programmes tended to assess progress in terms of activity, such as the numbers of vaccinations performed, rather than the result achieved	Clear, specific, and measurable goals drove a focus on results, with prioritization of quality measurement and verification over quantity	Set clear, measurable targets related to specific reductions in outcome metrics, rather than only measuring the number distributed
	Diversity of contexts and challenges meant no set checklist of methods for how vaccination campaigns and case finding should be carried out was possible	Experimental learning and avoidance of formalized programming facilitated identification of local solutions Problem-solver staff with reputations for adaptability, imagination, and hard work hired to serve as catalysts rather than controllers Hire and retain strong managers and operations officers to ensure execution	Hire flexible problem solving staff with backgrounds not limited to technical areas of malaria and public health Provide management training to programme leaders and aim for retention of strong managers
	Supervision was insufficient due to workloads and insufficient travel into programme areas to observe problems	WHO, national, and state or provincial supervisory staff encouraged to frequently travel into the field to review activities and work with field staff in resolving problems	Encourage managers from all levels to spend as much time as possible working in person with local programmes in endemic regions

### International support for the eradication programme

Henderson declared, “For a global programme against a disease to be undertaken, universal political commitment is necessary” [10]. In the case of malaria, support for fighting the disease seems strong, with malaria control activities frequently cited as one of the “best buys in global health” [11–13]. However, the pursuit of malaria eradication is more controversial, and whether it represents a feasible or even a worthwhile goal has been frequently debated [14–21].

Support for the smallpox eradication programme was similarly far from universal. The failures of prior eradication or regional elimination efforts including hookworm, malaria, yellow fever, and yaws increased skepticism, as did a perception that vertical eradication campaigns detracted from provision of basic health services [7]. Although the WHO was tasked with coordinating the effort from Geneva, its diverse departments and regional offices were not uniformly behind the effort, in part because “their officials competed with each other for finite financial resources and administrative influence” [22]. The Director-General of WHO reportedly had so little faith in the programme that he explained to Henderson—a secondee from the United States’ Centers for Disease Control and Prevention—that “he wanted an American as the director so that when the programme failed, as he was sure it would, the Americans, not the WHO, would be seen as responsible” [23].

The smallpox programme survived at the WHO in part because of strong backing from both the United States and the Soviet Union [23], the major powers of the era. Henderson himself was seen as a trustworthy leader by both rival countries despite ongoing Cold War hostilities because of his strong track record as an “honest and a good scientist” whose “only objective [was] to eradicate smallpox” [23]. Still, maintaining smallpox’s profile within the WHO and encouraging countries to contribute funding and resources was an ongoing challenge. Henderson used the annual meeting of the WHO assembly as an important opportunity to keep eradication on the minds of health ministers [8] and tried to maintain smallpox’s public profile by widely releasing surveillance reports with summaries of progress and problems. Henderson later suggested that a mistake he made was not adding dedicated staff to his team focused on public relations and donor advocacy [10]. Malaria today appears to have a more visible profile internationally than smallpox did, in part due to similar communications efforts, such as the annual World Malaria Report which provides opportunities for visibility and public engagement [24].

### International coordination of the eradication programme

International coordination was considered important to avoid “ping-pong smallpox” [25] in which infections would be continually reintroduced from country to country. A 1960 Inter-Regional Smallpox Conference organized by the WHO reported that since “the eradication of smallpox cannot be considered on the basis of individual territories,” the Conference “therefore urges the health administrations of all countries in endemic regions to synchronize their eradication campaigns” [26]. While this declaration was sufficient to spur action in some countries [27], others, including Brazil—the country with the largest burden in the Americas—and many African countries [28], declined to initiate vaccination programmes, compromising the possibility of regional success [29]. Provision of dedicated smallpox funding in 1967 proved critical to allow the WHO to incentivize countries to scale up their national programmes [10], even when committed funding was small [29]. The provision of donor funding for malaria—increasing from about $170 million in 2000 to $2.5 billion in 2016 [30]—has likely been similarly important to convince countries to prioritize malaria programming.

Despite the international push from the WHO, the smallpox eradication effort would always remain a collection of individual national programmes, each attempting to solve their own problems through their own systems and in their own ways [28], rather than a top-down, centrally managed global undertaking. Dr. William Foege, an American epidemiologist who helped design the surveillance-driven vaccination strategy that likely enabled success in countries including Nigeria and India [31], called it “20 programmes trying different things to more quickly discover truth” [32].

“The campaign to eradicate smallpox worldwide is often described in simplistic terms… The picture presented is of a unitary programme of action, where the many cogs in the wheel apparently worked in almost perfect harmony, causing orders from the top of an administrative pyramid to be unquestioningly implemented in localities across the globe… the organized drive to expunge smallpox was a much more complicated and disjointed entity” [33].

Current malaria guidance embraces an aligned belief that “adapting and tailoring interventions” to the local context will be important for elimination success [34]. While encouraging local solutions, the WHO and other international entities including the United States’ Centers for Disease Control and Prevention [35] added substantial value to these independent programmes, including:

#### Sharing best practices across countries

WHO’s guidance to countries changed substantially over the course of the programme as understanding of best practices evolved. Its initial recommendation for every country to vaccinate at least 80% of the population increased to a goal of 100% vaccination [28], before being replaced with a dramatically different recommendation to invest heavily in surveillance and to focus vaccination on the places where transmission was observed. Many countries resisted this latter change despite evidence that that surveillance-driven targeting was more efficient [31], and the WHO’s leadership in pushing for adoption of proven approaches was thus critical [36]. Today, regular revisions of malaria guidance (e.g., [34, 37, 38]) demonstrate that such dissemination of best practices remains an important WHO role.

#### Ensuring the quality of tools

Smallpox programmes relied upon having a stable, reliable, effective vaccine [39]. Yet when the newly established eradication headquarters in WHO established a system for testing batches of vaccine produced in more than 40 different countries, it found < 10% of samples were acceptable [40] due to potency and heat stability issues [41]. The WHO engaged vaccine experts to write simple manuals of production that explained best available production methods, and the WHO consultants worked with laboratories to improve their production processes [42]. Local production of vaccine was set up at government-owned facilities or associated institutes in the largest population countries including Brazil, India, and Indonesia, since donations would otherwise have been insufficient [42]. Two high quality laboratories from the Netherlands and Canada were selected to serve as vaccine reference centres [39], and they performed batch testing to evaluate improvements. As a result of these efforts, the fraction of batches meeting quality standards rose to 31% in 1967, 76% in 1972, and 96% in 1976 [36]. The WHO today provides an analogous quality control and assurance function for certain malaria commodities, prequalifying malaria drugs (https://extranet.who.int/prequal/), evaluating the accuracy of diagnostics [43], and inspecting manufacturing sites for vector control tools, though the complex landscape for malaria commodities makes it more difficult to assess the overall quality of the tools being used in endemic countries. The smallpox experience suggests that investment in the production of bed nets in high-volume countries could be considered as a possible means of reducing reliance on imported, donor-funded products [44].

#### Provision of technical and operational support

The WHO’s smallpox eradication unit provided national programmes with both field epidemiologists for technical advice and administrators to help manage logistics. Over the 12 years of the programme, 687 different individuals from 73 countries participated in the WHO-sponsored programme [45]. The expansion of WHO’s role from solely providing technical advice to actively enabling operations was a learning experience for the Geneva-based programme [46]. This evolution allowed Geneva to strengthen global logistics, moving supplies from one country to another as needed, or flexibly providing necessary funds to overcome bottlenecks [10]. It was noted that the WHO was most effective when its staff, including senior leadership, spent their time working in country with programmes [47]. Henderson stated his opinion that the most effective WHO staff “were those who took an active role in field operations. Those who assumed a passive role of detached technical adviser were encouraged to leave the programme” [10]. Similar sorts of temporary field advisors have been deployed under the “Stop Transmission of Polio” programme [48] and can prove useful for building capacity in malaria programmes if deployed thoughtfully [49]. The United States President’s Malaria Initiative today provides technical advisors to malaria endemic countries in this mode, as do several non-governmental organizations.

#### Encouraging research and innovation

In Henderson’s view, “The importance of problem-oriented research that was conducted throughout the course of the smallpox eradication programme cannot be too emphatically stated” [10]. Development of a heat-resistant vaccine may have been the single most impact factor in global success [7], while ongoing operational research enabled resolution of unforeseen challenges that inevitably occurred over the course of the long, complex undertaking of eradication [21]. The WHO encouraged such studies through its convening power [14], though innovation was typically decentralized. “An important lesson was that parallel activities and research, with many groups seeking better approaches, could speed up the process of improvement,” Foege wrote [50]. The jet injector, for example, a new tool for increasing the speed and efficiency of vaccine delivery [51], was first developed in the United States at the National Communicable Disease Center during the 1960s [52]. The development of a low-tech, simpler solution—the bifurcated needle—by a private company, Wyeth Laboratories (which waived patent costs for any manufacturer supplying them exclusively to the WHO [53]), proved both simpler [29] and ultimately more successful [54]. An examination of innovation in the smallpox programme concludes that what was important was to “insure that the problem has been defined clearly and that intervening variables and technological factors do not becloud that definition”, while building organizations that scientifically evaluate evidence and seek to improve themselves according to measurement of what does and does not work [39]. This perspective suggests the importance of continued investment both in malaria’s $540 to $600 million research and development pipeline [55] as well as in efforts to help countries collect, analyse, and apply data for ongoing organizational improvements within their own programmes.

In playing these roles, there was agreement that the WHO’s success was strongly linked to the ability to be as flexible and non-bureaucratic as possible [21]. Sometimes, as when flying to countries with outbreaks without receiving travel approvals, this meant breaking WHO rules [41], something Henderson deemed necessary given “a sclerotic… administration that often thwarted or actively impeded what appeared to be logical initiatives” [7]. In one example, an emergency request for vaccine supply from Uganda took 5 months to be transmitted to headquarters by the regional WHO office, during which time the Geneva office had already learned about the outbreak via informal backchannels and addressed it [8]. Internal WHO disagreements also led to challenges, with Henderson noting, “Officials located within different levels and departments of the regional offices continued to hold disparate views right till global smallpox eradication was formally certified” [33]. He complained that, “The regional offices of WHO… were more a hindrance than a help,” leading him to adopt a “policy of quietly short-circuiting the regional office, when necessary” [8].

The challenge for a complex bureaucracy like WHO to nimbly respond to dynamic circumstances have been echoed in recent years by criticism surrounding its response to the 2013–2016 Ebola outbreak in West Africa [56, 57]. The success of the WHO’s smallpox team may provide a model for how a Geneva-based team can flexibly facilitate malaria operations across endemic countries. However, the fact that Henderson and colleagues viewed their success as something they achieved despite WHO’s structures and procedures—for example, by creating a new unit within a regional office that reported directly to Henderson rather than through the normal channels [33]—rather than because of them, suggests that consideration will need to be given to how to ensure a central malaria coordination team is encouraged and enabled to be agile and flexible, as is required by the rapidly evolving nature of a global eradication enterprise, while still respecting and sometimes deferring to local solutions and expertise.

### Financing the programme

Achieving malaria eradication will require each of the world’s endemic countries to invest in eliminating transmission. Financial analyses typically suggest that substantial short term budget increases will be required to end endemic transmission, after which long term savings can be realized due to the lower costs of preventing its re-establishment [58, 59]. Surprisingly, in the case of smallpox, Henderson argues no such surge in funding was required, with existing domestic budgets sufficient to cover programmatic needs:

“The burden of expenditure has been borne by the endemic countries themselves… But, with few exceptions, the expenditure by the countries has been little more than what they were already spending to *control* smallpox. In other words, WHO and its member countries, with only a very modest additional input in resources, have transformed a never ending control programme to a successful *eradication* programme.” [29]

The idea that smallpox could be eliminated from countries with essentially the same budget previously used to control it is remarkable, and suggests that how funds were spent proved far more critical than the total amount of those funds. As Henderson describes:

“For all of us it has been a revelation in so many countries to find at the periphery such an array of unproductive health staff and facilities. It has been a revelation to discover how effectively they may be mobilized with a comparatively small input involving leadership in the field and definition of a series of activities with defined objectives and a modest element of management. Other health programmes, especially those involving immunization, but others as well, could, I believe, be similarly transformed.” [29]

The importance of using available funding better was raised both nationally and internationally. The WHO internal dynamics and disagreements between regional offices complicated the efficient expenditure of available funding. In the Americas, for example, in the early 1960s, the Pan American Health Organization (PAHO) chose to distribute available funding for mass vaccination across the entire region, even though Brazil was the only remaining endemic country [6]. As a result, Brazil’s funding was insufficient and elimination programmes were prolonged unnecessarily [14]. The WHO’s South-East Asia Regional Office (SEARO) chose to pass up the available funding rather than participate in the programme, which it disagreed with; Henderson then channeled the SEARO money to PAHO in hopes it would be spent in Brazil. Less than half actually was, with the remainder divided across 10 other countries [8]. When 5 years later Brazil was finally free of smallpox, PAHO refused to donate its funds back to SEARO in turn to assist India [8].

Henderson’s comparison of the relatively similar costs for control *versus* eradication refer only to domestic contributions, and do not include the 407 million doses of vaccine that were donated over the course of the programme, primarily by the Soviet Union and the United States [29], at an average estimated value of $17 per 1000 [6] (approximately $7 million in total). Between 1967 and 1979, $67 million in cash and kind (including the donated vaccine) was donated to the WHO’s special account for smallpox eradication while $33.6 million was spent from WHO’s regular budget [6]. This total of approximately $7.7 million per year would translate to approximately $30–$50 million in today’s dollars—far less than the $2 billion per year currently contributed by international donors to malaria programmes [60].

The argument made to donors to secure these funds was that “all should be willing to contribute to carry the attack to the remaining endemic regions until there is no more smallpox” [51]. The United States, for example, was said to be domestically spending $140 million annually in 1968 to prevent re-establishment of smallpox transmission domestically, and thus its modest investment of $15 million to eliminate in West and Central Africa meant that it could help 20 countries become smallpox free for the price of 39 days of preventing its reintroduction back home [35]. A similar argument was used to successfully convince the Swedish government to make a critical contribution to the programme in India, since “every country is in danger until the last case of smallpox has been eliminated” [22].

The availability of even small amounts of funding that could be used flexibly, with minimal bureaucracy, was seen as critical to bypassing bottlenecks. “It was essential to have an allocation of funds that could be used for any necessary purpose and in any country” [10], yet nearly all available funds for smallpox eradication were earmarked for specific uses. As a result, staff were often not paid on time, insufficient fuel allowances meant vehicles were not available when needed, and funding for car repairs was lacking in multiple countries [10]. In Zaire, for example, operations would frequently grind to a halt after the government failed to release the necessary funds; the programme solved the issue by setting up an auxiliary bank account in which they deposited back-up funds whenever possible to cover expenditure during these gap periods [8]. In Bihar, India, “staff were fearful of paying too much [for vehicle maintenance] and being held accountable for extra charges” [46], so vehicles were often neglected instead. New accounts were set up to give team leads advances for these minor but essential charges so that they could avoid weeks of paperwork to receive necessary funds, instead providing receipts at subsequent meetings on a biweekly or monthly basis. This approach dramatically improved the flexibility of the elimination efforts and Henderson deemed it “one of the most important initiatives of the programme” [8].

Malaria programmes today frequently experience similar delays due to challenges with financial expenditure. Many countries have failed to spend grants from the Global Fund to Fight AIDS, Tuberculosis, and Malaria on schedule due to a wide variety of issues, including lack of human resources, delays in procurement, weak data systems, and other challenges [61]. The smallpox experience suggests that the proactive creation of a flexible fund that could be used to address bottlenecks across countries as they arise could be a valuable tool for malaria as eradication proceeds, though the challenges of ensuring those funds are well spent would be substantial, and safeguards would be needed to ensure funds are spent for their intended function. This history also emphasizes the critical importance of having strong measurement and management of programmes to ensure available funds are allocated and used as effectively as possible.

### Domestic support for the programme

Political will has been cited as one of the most important factors in the success of smallpox eradication [14] and a necessity for eliminating malaria [34]. Not all countries viewed smallpox elimination as an urgent priority given many other public health issues [28], just as malaria elimination is often a low priority today for countries facing more visible threats [62]. Competing disease priorities, including ongoing malaria eradication efforts [29], led governments such as that of Ethiopia to have “absolutely no interest in the eradication of smallpox” [63]. Non-governmental actors such as the United Nations Children’s Fund (UNICEF) also had prior commitments to malaria eradication that took precedence over contributions to smallpox [8]. Today, the need to devote substantial resources to ongoing efforts to eradicate other diseases, including guinea worm and polio, may present similar challenges for malaria.

Countries where the less virulent variola minor predominated over the far more deadly variola major, mostly in Africa, tended to downplay the importance of embarking on an elimination programme, given that this strain of the disease was “little more serious than chicken pox” [8]. Henderson cited this reticence as one of the two primary factors compromising the young programme (the other being the absence of funding) [10]. This challenge is echoed by questions of whether malaria eradication should aim to include all species of the disease or only (or initially) the more virulent *Plasmodium falciparum* given its outsized contribution to mortality as well as its development of resistance to artemisinin-based drugs in the Greater Mekong subregion [64, 65]. Accounts of smallpox eradication do not clarify whether an effort to only eradicate variola major could have succeeded (and thus whether a *P. falciparum*—only attempted might be feasible), though the similarity of symptoms between the two would have complicated case finding directed only at the major variant.

Political backing also suffered with changes in government and thus the loss of advocates: “Within 4 years after the West African programme began, there were 23 changes of governments in the 18 participating countries,” causing “changing leadership and staff in the nation’s smallpox programme” [47]. In India, it was noted that the Prime Minister’s enthusiasm for smallpox typically increased when outbreaks were observed—and thus when the electorate was most concerned about the disease—and declined with smallpox incidence [22]. Pressure from powerful allies outside the government was thus seen as critical to ensure the programme remained sufficiently well supported even when smallpox was not in the headlines. An agreement to begin a vaccination programme in Ethiopia only occurred due to the intercession of a senior Austrian physician with a close relationship with the Emperor [63], while in India, the intervention of J.R.D. Tata, the well-connected head of a large corporation, played a critical role in convincing the Prime Minister to continue supporting the smallpox programme at a pivotal moment [22]. In Bhutan, where the WHO initially lacked visibility into smallpox efforts due to the secrecy of its government, an acquaintance of Henderson’s with access to the royal family was eventually able to build communications with Geneva [66].

A lesson for malaria is thus the importance of getting well-connected leaders from business and high-profile institutions to act as advocates. The opinion of politicians can change based on what seems important for the next election, but smallpox programme examples show how they can be convinced by counsel from those they trust or respect. Malaria appears to already be doing a better job of identifying high-profile advocates; organizations with the explicit goal of maintaining malaria’s global or regional visibility, such as Malaria No More or the African Leaders Malaria Alliance, identify champions who can contribute funding and political backing to national efforts [67], while the End Malaria Council (http://endmalariacouncil.org/) seeks to bring business leaders together with public sector leaders to keep malaria a global priority.

### Community support for the programme

Community participation with the smallpox programme was considered generally strong [8], although the literature contains numerous accounts of specific anecdotes of resistance to vaccination particularly following real or perceived adverse reactions to the vaccine [28]. Some commentators note that the narrow focus on smallpox was sometimes counterproductive given the range of health issues afflicting communities. In Bangladesh, for example, vaccination occurred in the midst of a cholera epidemic, yet the vaccinators could provide no assistance with the more visible and urgent problem, resulting in community frustration [68]. As the programme proceeded, additional components were therefore added onto the responsibilities of surveillance agents to keep them engaged and motivated despite the infrequency with which smallpox was observed, including surveys investigating access to clean water, vitamin A, family planning, and rates of childhood mortality [68]. Similarly, malaria-only health workers may prove less successful than those that have been trained to treat a variety of common illnesses [69].

Gaining the support of community leaders was commonly cited as a crucial step towards community acceptance. In Nigeria, Foege believed that people participated less because they were convinced by vaccinators to do so and more because they trusted their leaders [31]. In one extraordinary case, vaccinators were reported to have awed a village chief into supporting the programme by releasing a trained bird to swoop overhead and drop pro-vaccine leaflets while vaccinators were meeting with him [29]. Despite such anecdotes, Tarantola and Foster note that little research was conducted into how the community could best be engaged [68], though attempts to do so included deployment of midwives and other village workers to engage and educate the community [39, 70] as well as provision of monetary awards for report of a smallpox case in the final stages of the programme [71]. In India, for example, a 100 rupee reward was offered for anyone reporting a previously unknown outbreak [29]. The evidence base for what drives patient participation with the health system has increased in subsequent decades, identifying factors related to cost, proximity, and confidence [72], but the relative ability of different interventions to influence those factors likely still requires additional research. Best practices for proactive engagement of community leaders and ongoing communication and collaboration with at-risk populations should be encouraged to make communities active participants in malaria elimination programmes [73].

Where efforts to improve participation failed, smallpox programmes would sometimes use compulsory vaccination, an approach that dispensed with “the need to converse with villagers at all” [74]. Compulsory vaccination was believed to be justified by the need to achieve sufficient coverage for the greater good, but it raises troubling ethical questions. Greenough quotes Stanley Music, an epidemiologist who worked in the Bangladesh programme, on the tactics sometimes employed:

“In the hit-and-run excitement of such a campaign, women and children were often pulled out from under beds, from behind doors, from within latrines, etc.… Attempts were made to secure the cooperation and ‘blessing’ of village headmen, thereby putting social pressure on the villagers to stand their ground and accept vaccination. Still, however, some form of minor chaos was the rule, as headmen’s authority did not extend into individual’s homes… People were chased and, when caught, vaccinated… We went from door to door and vaccinated. When they ran, we chased. When they locked their doors, we broke down their doors and vaccinated them.” [74]

While these aggressive approaches did in some cases attain the narrow goal of achieving high vaccination coverage, they seem unwise for a programme such as malaria in which long-term participation and repeated delivery cycles is needed. Ethically, they were controversial even at the time, and “the organized and sustained use of compulsion was, generally speaking, instituted with great care and only after broad administrative and political consensus had been achieved” [22].

Engagement with the private sector was reported to be generally minimal outside a few efforts to integrate private health care providers into the vaccination programme [68]. India proved one of the main exceptions, with the Tata Group playing a critical role in vaccinating the population of Bihar State, where its steel plant was located. They provided “medical and paramedical personnel, transportation, managerial support and communication facilities to implement the programme activities. The assistance in kind provided by the Company and their local knowledge of the area were so valuable that south Bihar became smallpox-free in a record period of 6 months” [75]. Malaria’s recent history includes several examples of similar partnerships [76]. Given the importance of private providers and drug shops for provision of malaria treatment [77], malaria eradication will necessitate much greater engagement with the private sector than occurred during smallpox eradication.

### Programmatic strategy

Smallpox eradication was predicated on the idea of mass vaccination of the population. The WHO’s Expert Committee initially called for countries to achieve at least 80% vaccination of the population [29]. This approach successfully led to elimination in some countries, but elsewhere it failed, likely because the vaccinated and unvaccinated fractions of the population were not homogenously mixed [36]. In Central Java, for example, a 1969 survey found greater than 95% vaccination rates had been achieved across the population of 23 million people, yet that same year over 1700 cases were recorded, nearly all amongst the 5% of the population who had been missed [78]. The WHO Expert Committee responded by telling countries they should strive for 100% vaccination rates, a target scorned as impossible [29]. Attempts to conduct greater numbers of vaccinations were undertaken, but “accessible groups, like schoolchildren, were vaccinated repeatedly so that high ‘scores’ were achieved, but there always remained a large pool of unvaccinated persons” [47]. This language is mirrored in a recent investigation of bed net coverage across Africa by Bhatt et al., which concluded:

“We found substantial over-allocation of nets to households already owning a sufficient quantity… What is certain is that over-allocation becomes a major barrier to achieving universal coverage when levels of [insecticide-treated bed net] provision are high because most new incoming nets are simply leading to surpluses in many households, while elsewhere there remains a shortfall. This may have a disproportionately high public health impact if those surplus nets are concentrated in households at lowest risk.” [79]

The critical change in smallpox programmes was a shift away from mass vaccination towards an approach called “surveillance-containment” [35] in which programmes sought out smallpox cases and then concentrated vaccination efforts in their proximity and towards those who may have come into contact with the cases. In short, the new strategy meant focusing vaccination on the places where it was most likely to matter, rather than laboring to achieve implausibly perfect coverage everywhere. In Bangladesh, for example, the programme successfully ended transmission after abandoning efforts to achieve 80% vaccination nationally and focusing efforts instead only on the northern districts where cases were reported [41].

The 1964, the WHO Expert Committee report did not even mention surveillance [8], but the new focus on finding cases, tracking down all of their contacts, and concentrating vaccination operations in the most necessary places was considered by many to be one of the keys to eradication’s ultimate success [14, 80]. Identifying where smallpox was being transmitted required a network of agents who visited all health units (usually in teams of two to four per administrative unit) to ensure weekly reporting, sought out cases in the community, including by collaborating with teachers or visiting markets [78], and distributed surveillance reports so that the health staff saw how their reports were being used [81]. “Undoubtedly, the greatest stimulus to reporting was the prompt visit of the surveillance team for outbreak investigations and control whenever cases were reported,” Henderson wrote. “This simple, obvious and direct indication that the routine weekly reports were actually seen and were a cause for public health action did more, I am sure, than the multitude of government directives which were issued” [81]. Case finding was intensified during the period of lowest seasonal incidence, since that low transmission season represented the weakest point in the smallpox cycle and the best opportunity to break transmission, despite the operational challenge of finding cases at that time of year [82]. Active case finding was integrated with routine reporting from public health facilities rather than conducted entirely in parallel [81]. Challenges to setting up good surveillance systems included the fact that in many countries, disease reporting fell under the purview of independent statistical units and were not thus within the control of the smallpox programme [10] (the same is true for malaria today in many countries).

The operational strategy of directing vaccine only to known transmission areas may not be directly translatable to malaria’s tools. First, the approach may have worked in part because the reproductive rate for the virus was relatively low [35], estimated at approximately 3.5 to 6 [83], while estimates for malaria are variable but potentially far higher [84]. Second, case finding was far easier because the symptoms of the disease were so distinctive and recognizable even to schoolchildren [81], and smallpox—unlike malaria [85]—very rarely caused asymptomatic infections [6]. As a result, mathematical modeling of an analogous reactive case detection strategy for malaria suggested that such approaches may increase the probability of elimination in certain contexts, but would be “a highly resource intense, long-term intervention that is inappropriate in many settings where resources are limited” [86].

Nevertheless, the critical shift in smallpox programmes from judging success based on the volume of vaccinations to whether vaccination was achieved in the most necessary places still suggests a good model for malaria programmes, despite the extensive presence of asymptomatic carriage. Malaria programmes that seek only to distribute commodities such as nets or drugs in high volumes in an attempt to achieve “universal” coverage may be missing more inaccessible populations which may also be the highest risk for malaria [79, 87]. Shifting towards a risk-focused approach in which prevention and treatment are targeted to those who most need them has great potential for improving the efficiency and effectiveness of our efforts.

### National programme structure and management

Discussion of the wisdom of eradication programmes often revolves around the relative merits of “vertical,” single disease programmes *versus* “horizontal” health systems efforts [14], which were increasingly coming into favour at the WHO around the time of smallpox eradication. Henderson advocated for having a specific vaccination programme distinct from, yet linked to, routine health services, worrying that fully integrated programmes would lack clear objectives, evaluation systems, and management structures. “The ‘horizontal programmes’ I have seen best describe the sleeping postures of the workers” [80], he wrote. In contrast he considered a “targeted and time-limited special programme with funds specially allocated for it, both in the WHO budget and in most national budgets, and with full-time technical staff responsible for its supervision” [10] to be superior since it would more easily attract resources and community support and likely be more efficient and better managed given the singular focus. Such programmes were also viewed as attractive because they could be conducted even while basic health services remained weak [51]. The vertical *versus* horizontal health programme debate has persisted since smallpox [88] and will not be resolved here, yet a few clear lessons for malaria emerge from smallpox’s successes.

First, smallpox programmes were well integrated with basic health systems, enabling routine case management and surveillance, with active case finding used as a supplement rather than a replacement. This integrated design improved upon the design of the Global Malaria Eradication Programme preceding it in the 1950s and 1960s, which largely circumvented basic health systems. The malaria eradication programme measured malaria primarily via population prevalence surveys [89] and other active means [90] and conducted insecticide spray campaigns as vertical efforts. Malaria staff were also better paid than other workers and reported to heads of state rather than ministries of health, creating unsustainable systems [7]. In contrast, smallpox programmes were still part of the health system, leveraging the same basic health services and staff to identify and report the disease [10, 33]. This integration meant that smallpox teams were not required to set up fully parallel surveillance systems, instead augmenting existing ones and leaving behind some added capacity within health programmes. Similarly, reliance upon the routine, albeit imperfect, measurement of malaria that basic health systems provide across endemic regions seems likely to greatly improve the cost-effectiveness of surveillance given that it requires minimal expenditure beyond keeping health facilities stocked with diagnostic tests, training staff in their use, and linking them to effective reporting systems. Such investment in core case management systems is a primary component of WHO’s Global Technical Strategy for malaria [4].

Second, multiple authors highlight the importance to smallpox programmes of creative, problem-solving staff [32] who could figure out how to overcome any obstacle that arose, tailoring solutions to the unique challenges and contexts faced by each country [10]. Henderson described:

“The essence of what has made the programme what it is is, very simply, an imaginative and dedicated field staff, both national and international, who, given scope and encouragement to work out problems according to local circumstances and support in their efforts to do so, have responded with some remarkable solutions to impossible problems.” [29]

These resourceful workers, described by former United States Surgeon General Julius Richmond as “simply too young to know it couldn’t be done” [50], were supported by a similarly flexible international team at WHO, who were described as:

“Essentially problem-solvers, they viewed themselves as catalysts rather than as controllers. They understood from the onset that experimental learning offered the only possibility for success. They avoided formalized programming, opting instead for innovation, flexibility, communication and experiment, by means of a number of deliberate policies and mechanisms. They recruited people with practical field experience in epidemiology (as opposed to previous work with smallpox per se). They sought people with reputations for adaptability, imagination, and hard work. They preferred younger people, assuming they would be more receptive to new approaches and ideas.” [91] quoted in [39]

Henderson contrasted the flexibility with which smallpox programmes worked with the unsuccessful prior malaria eradication effort, which he said “was conceived and executed as a military operation to be conducted in an identical manner whatever the battlefield” [92], preventing it from adapting to local contexts, structures, and systems.

Third, smallpox programmes placed great emphasis on careful measurement and verification. “Logic suggests that all disease control programmes should provide continuous measurements of disease incidence, and that these measurements should dictate changes in strategy and tactics,” wrote Henderson. “In fact, few programmes do so. Responsible authorities tend to ignore such information or dismiss efforts to obtain the data and, instead, assess progress in terms of activity, such as the numbers of vaccinations performed or patients treated” [10]. Arguably, today’s malaria programmes continue to focus more on activities conducted rather than impact, in part because key performance indicators reported as proof of performance on grants such as those from the Global Fund to Fight AIDS, Tuberculosis, and Malaria tend to focus on the number of nets delivered [93], rather than whether they are delivered to those most at risk or achieve desired reductions in malaria. The smallpox experience suggests that successful elimination may require shifting focus from simply tallying how many commodities have been distributed towards assessment of whether those tools are being used as effectively as possible.

In West and Central Africa, smallpox programmes used three different types of evaluation approaches: first, evaluators would follow-up to assess whether what vaccinators claimed to have done had truly been accomplished; second, tally sheet comparisons were made to compare vaccination records against any available census data, as a quick if somewhat inaccurate estimation of whether numbers were approximately what should be expected; third, spot checks for vaccine scars were conducted at markets and other convenient gathering places to provide an independent confirmation of coverage [94]. Henderson stressed that in measurement, quality was more important than quantity: “a few indicators of overall performance, closely followed, were more useful than a broad spectrum of indicators measuring many aspects of programme execution” [10].

How to build appropriate teams and processes to conduct this measurement and verification was determined on a country by country basis. In Bolivia, one inspector was appointed for every eight to 12 vaccinators, ensuring everyone’s work was reviewed at least biweekly [95]. In India, a Central Appraisal Team oversaw evaluation processes, including frequent travel to trouble spots to assess what was going wrong [8]. Ensuring accurate reporting was sometimes compromised when workers avoided reporting true cases because they thought they would be punished for allowing transmission in their region [70], underscoring the importance of clear and frequent communication between central and local levels, with regular meetings to discuss problems and progress [10]. Widespread distribution of smallpox indicators was encouraged, such as through surveillance bulletins in Brazil which were distributed on a monthly basis to a wide audience, providing updates on progress, putting pressure on non-reporters to participate, and generally helping to foster a shared sense of purpose across the diverse network of individuals participating in the campaign [8].

Fourth, the smallpox programme emphasized the importance of strong management in all aspects of the programme. Henderson suggests that, “Successful execution of the programme consists of perhaps 10% technical skill and 90% organization and leadership” [29]. He stressed the importance of leaders actually spending substantial time out in the villages where the work is being done, leading by example and helping motivate workers: “effective leadership to solve the problems faced by field workers cannot be supplied by an army of physicians and senior supervisors who never leave their desks. Regrettably, these types are all too plentiful throughout the world” [29]. These opinions were substantiated by an evaluation of unsuccessful programmes in India, Pakistan, Argentina, Iran, and Ghana, which found that:

“First and most important, failure appeared to be associated with inadequate supervision and assessment. Programmes that failed normally showed the following shortcomings: (a) supervisory personnel did not check at the family level to assure that broad overage by vaccination of the population was being achieved; (b) supervisors were too burdened by other responsibilities to give more than nominal supervision; (c) inadequate provisions for travel and expenses; and (d) disinclination of supervisors to undergo the inconvenience of field work.” [22]

William Foege described how “the real problems” of “developing routines, documenting the implementation of those routines, hiring the right people, supervising, motivating, and evaluating” required “managers, administrators, and logistics experts—people who knew how to solve problems and how to get things done. The programme would not fail for lack of scientists, but it could fail—even with the best strategy—if we didn’t attract the very best managers” [32]. Strong management was required to keep up staff enthusiasm for searching for smallpox when there was nothing left to find [22]; in one case, near the very end of the programme in Ethiopia, a surveillance agent walked for 15 days to check on two reported cases which turned out to be chickenpox [29].

Programmes accordingly sought to hire non-medical, logistics-oriented staff with experience in administration in addition to those with a more conventional public health background [82]. Once brought into the programme, strong managers had to be retained: in Brazil, for example, five different directors were appointed in the 5 years between 1967 and 1971 [14] with unsurprisingly weak results. Henderson suggested that providing programme leaders with management training would have been a wise idea, though it was not done at the time [10].

## Discussion

The successful eradication of smallpox holds many lessons for malaria eradication efforts, despite the considerable differences between the programmes. Smallpox succeeded as a collection of individual country programmes each deriving local solutions to local problems, yet with an important role for WHO and other international entities to facilitate and enable these efforts by ensuring the best possible tools were available, maintaining the disease’s profile globally, fundraising, and arm-twisting in reluctant countries to ensure coordinated action. The documented experience of smallpox programmes suggests that such coordinating efforts must be nimble and flexible to stay relevant to rapidly changing country situations, and burdensome bureaucracy must be avoided if international agencies such as WHO are to add value rather than increasing the challenge of disease elimination.

Smallpox programme leaders stress the importance of empowering countries to solve problems locally. Where a strategy or tool has been proven to work well, efficient mechanisms for sharing those experiences are essential. Yet each country will need to adapt those effective approaches given their diversity of populations, systems, strengths, and weaknesses. Global leadership for malaria eradication must ensure countries are able to access the most effective tools available and understand the best principles for how to use them, but the smallpox experience suggests there is no script to be followed in elimination, no simple set of check-boxes that if ticked will result in success. Countries did benefit from the provision of international technical advice and logistical support, helping build staff capacity. The particular importance of administrative support to national programmes suggests distinct cadres of staff can add substantial value to malaria elimination programmes: advisors from public health backgrounds can help with technical aspects, but logistical experts are needed to help plan and execute efficient operations. The smallpox experience also emphasizes the critical importance of hiring programme leaders and managers who are enthusiastic about spending time with communities and local programmes, and who are creative thinkers who can derive context-appropriate solutions to the challenging problems that will inevitably arise.

Smallpox eradication is reported not to have involved a substantial increase in domestic budgets, but rather was achieved by better managing programmes and streamlining how they spent the available funds. A clear lesson is that data-driven approaches that target resources to the places where they are most needed will be more successful for elimination than mass attempts to achieve universal coverage everywhere; such a shift in mindset proved similarly successful in the eradication of rinderpest, with surveillance-targeted vaccination proving much more impactful than total coverage [96]. Minimizing inefficiencies in malaria programmes to ensure available funds have the greatest possible impact should be a high priority, even while the malaria community continues to advocate for increased funding from donors. In addition, the importance of flexible funding—even in small amounts—was repeatedly stressed. Setting up a central malaria account that can be rapidly and flexibly used for filling gaps and bypassing bottlenecks could be an important step towards enabling malaria eradication. Means of reducing dependence on donor-funded commodities, such as investment in local manufacturing, may also need to be considered.

Building a malaria elimination programme that is visible for fundraising and that has its own discrete, measurable milestones will drive programmes to hold themselves accountable and focus on achieving results rather than just distributing commodities. However, nesting those programmes within basic health services is critical to leverage routine case management and reporting, increasing the sustainability and reach of the programme. While government programmes may direct the fight against malaria, the experience of smallpox eradication also suggests affected communities and the private sector will have critical roles in whether success is achieved.

An innate limitation of this review is that it depends upon the published literature, which is constrained by the availability of viewpoints of those who have published [66]. Smallpox was a global undertaking with diverse contributions of healthcare workers at all levels of international and national programmes, yet accounts in the literature are primarily written by director-level staff from the United States and Europe. Accordingly, this review is biased substantially towards the viewpoints of those few individuals who dominate the literature.

## Conclusions

In Henderson’s view, many of the political challenges to eradication were unforeseeable, and ultimate success required luck as much as careful planning: “Had the effort begun a year earlier or later, it might have failed… In almost every country there were periods when neither surveillance nor eradication programmes were possible. The success of these national programmes often hung by a thread” [47]. Those involved in smallpox eradication disagreed about the right approach—and indeed whether eradication was even feasible—until the very last case [14]. Uniformity of opinion will similarly not be essential for malaria eradication to be successful. What is instead required is for national programmes and the international institutions that support them to be scientific in their approaches and efficient in their execution—to be open to new tools and strategies, to weigh evidence, revise approaches, and to make data-driven decisions as best they can given imperfect intelligence.

## Data Availability

Not applicable.

## Abbreviations

PAHO: The Pan American Health Organization
SEARO: WHO’s South-East Asia Regional Office
UNICEF: The United Nations Children’s Fund
WHO: The World Health Organization
